# Supplementary material for: RtcB2-PrfH Operon Protects E. coli ATCC25922 Strain from Colicin E3 Toxin
Source: Int J Mol Sci. 2022 Jun 9;23(12):6453. doi: 10.3390/ijms23126453 (PMC9223846; doi:10.3390/ijms23126453)
Supplement: Supplementary file 1 [file ijms-23-06453-s001.zip › ijms-1715667-supplementary.pdf]

International Journal of
Molecular Sciences

Supplementary Material

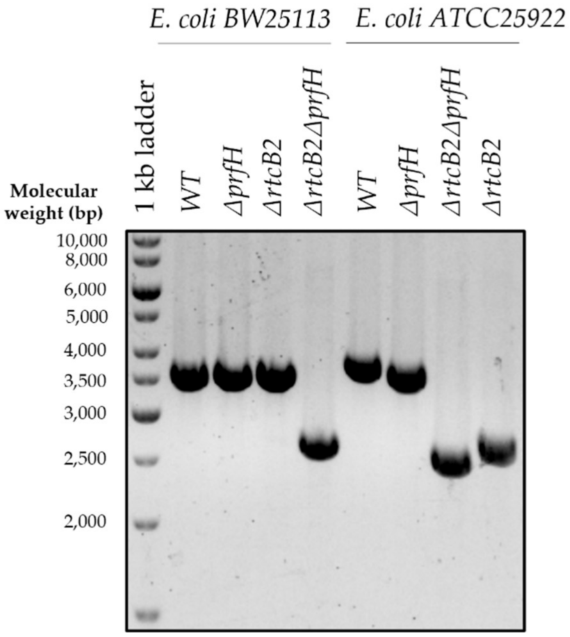

Figure S1. An agarose gel electrophoresis showing a PCR verification and confirmation for kanamycin sensitive strains, for both wild-type and knockout strains generated and, used for this study.

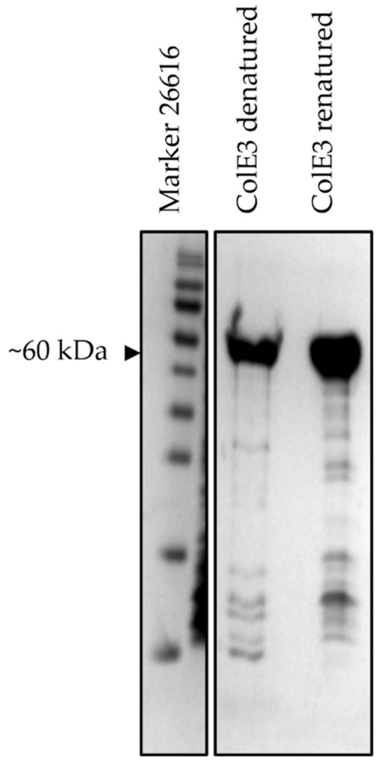

Figure S2. Colicin E3 purified under denaturing conditions. The isolated protein was approximately 95% pure after an extensive renaturation process in MilliQ pure water.

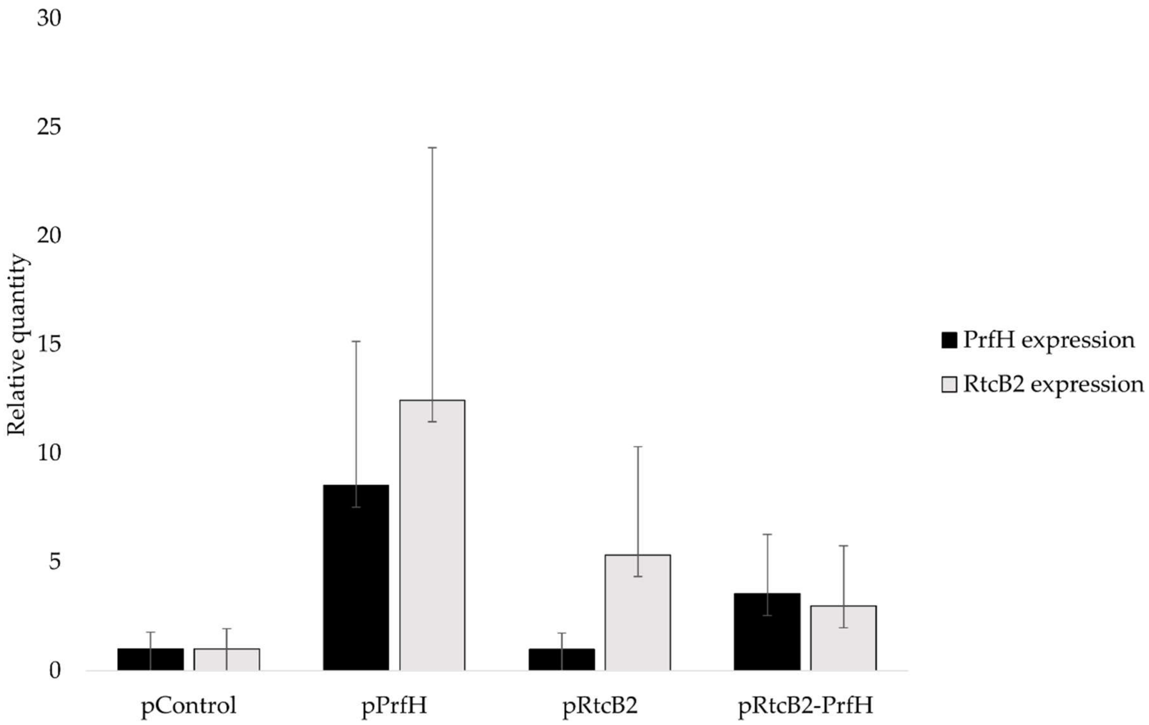

Figure S3. qRT-PCR analysis for the *E. coli ATCC25922 ΔrtcB2ΔprfH* complementation treated with colcin E3 protein. The elongation factor G (EF-G) gene was used as a housekeeping gene, while primers specific to *prfH* and *rtcB2* were used to score for the target genes respectively. The overall treated samples were normalized across all replicas, in order to calculate $\Delta\Delta C_q$ expression, relative to their corresponding untreated samples. Thereafter, the relative quantitation was scored relative to the control for test samples under investigation. The experiment was executed in triplicate. Error bars represent the overall standard deviation deduced from the relative ratio for ΔC_q expression between treated and untreated samples.

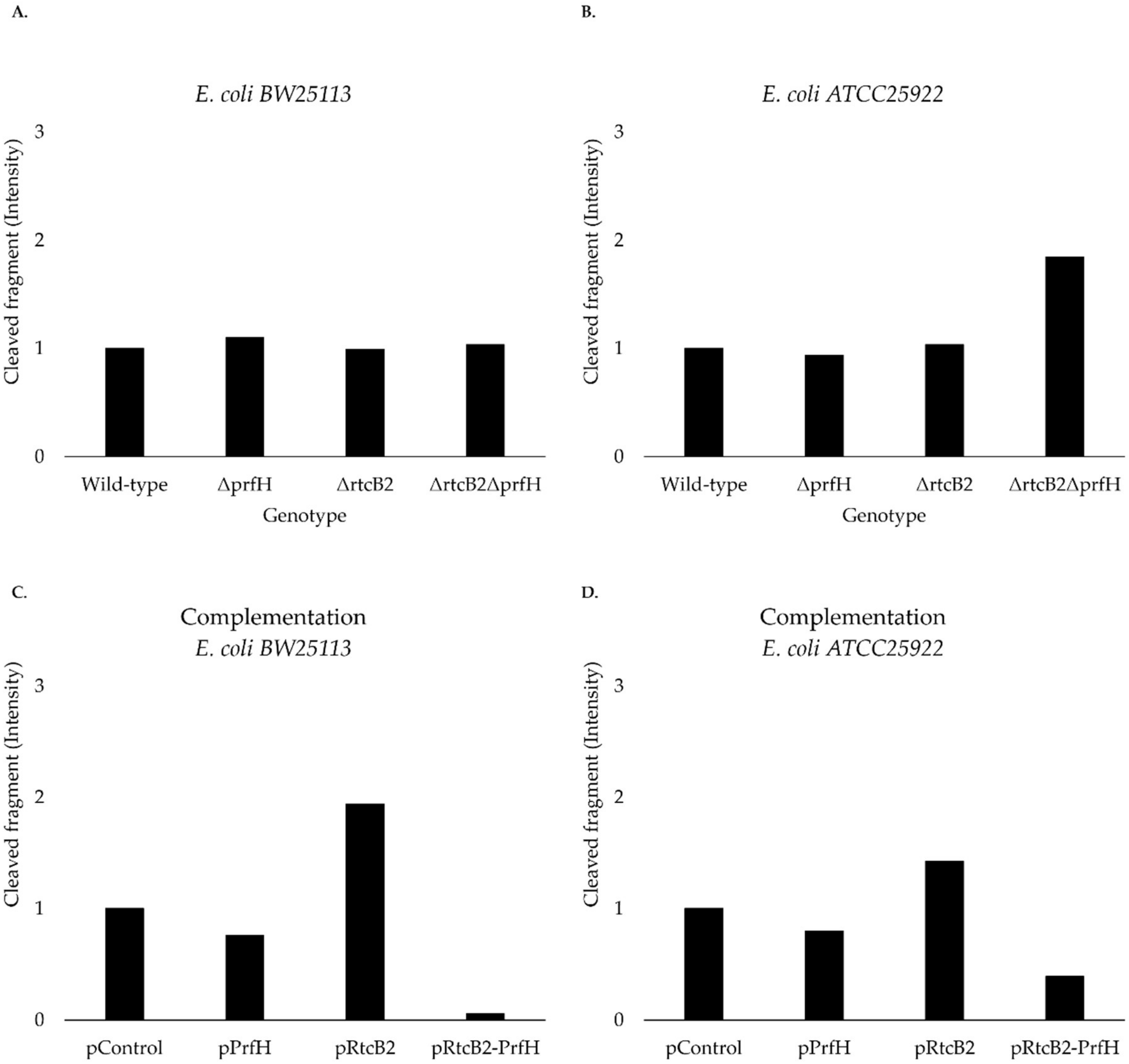

Figure S4. Quantitation of the colicin E3 cleaved fragment (~50 nt) bands from the SYBR™ Green II RNA-stained gel, scanned and scored using the ImagLab software (Bio-Rad). The intensity scored was normalized relative to the to either wild-type strain for (**A**) and (**B**); or pControl for (**C**) and (**D**) respectively.

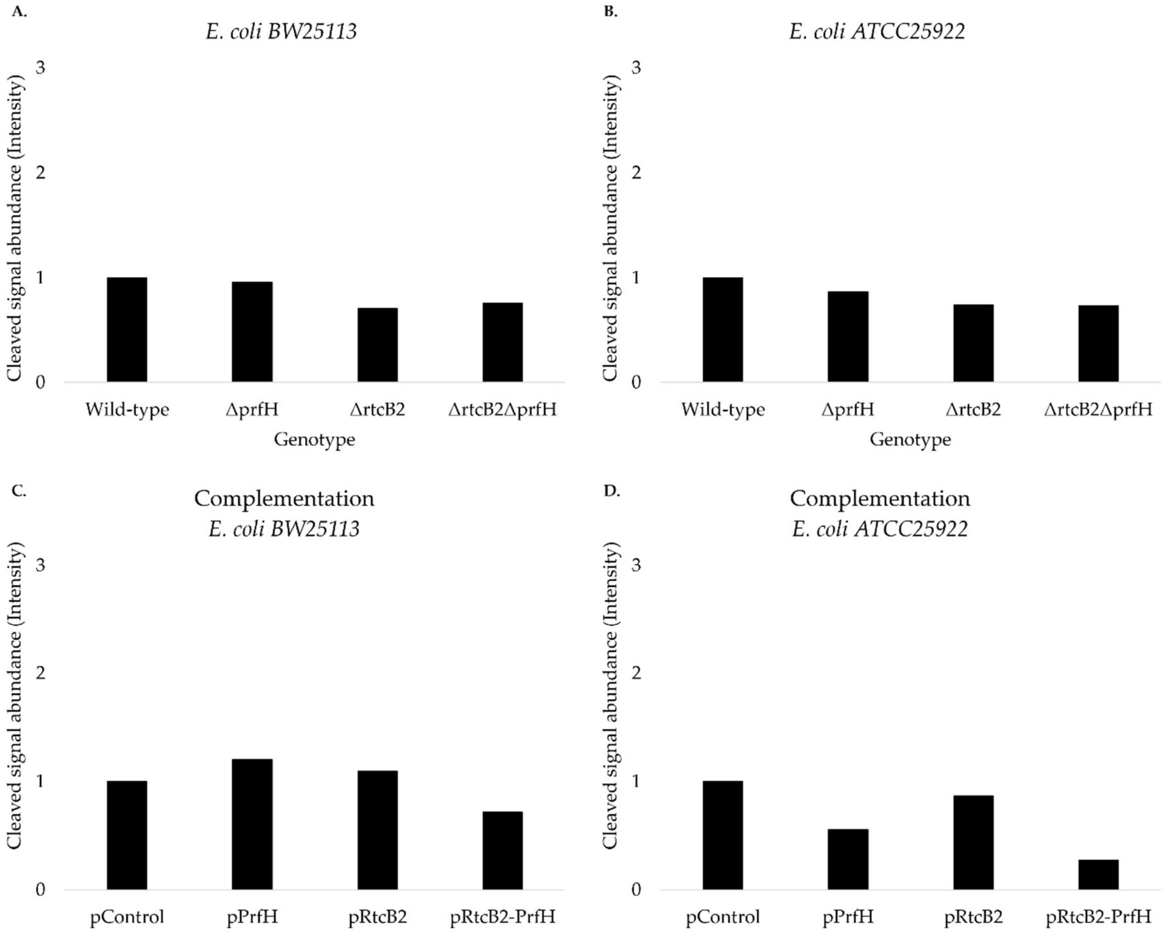

Figure S5. Quantitation of the colicin E3 intensity scored by determination of lanes corresponding to cDNA products synthesized by AMV reverse transcriptase using a γ-[32P] ATP labeled reverse primer complementary to the 16S rRNA. The ImagLab software (Bio-Rad), was used to decipher the total intensity of each respective lane which corresponded to samples treated by colicin E3. The intensity scored was normalized relative to the to either wild-type strain for (**A**) and (**B**); or pControl for (**C**) and (**D**) respectively.

Table S1. Primers used for recombination.

Number	Primer name	Primer sequence
1	F_H1P1_rtcB2_KO_FRT	ATGCGATATAAACCGCAACATTAAATCCAGCTTGCAATGAAAATAACGCCGTGTAGGCTGGAGCTGCTTC
2	R_H2P2_rtcB2_KO_FRT	CCCCTGAGCAGAGGAGAGTTGTAGCAAGATCATCCTTTTTTCCCTCCACTTCCTCCTTAGTTCCTATTCC
3	F_H1P1_prfH_KO_FRT	CGACTGCGCCCGGTGCTGACGCTCAAAAACAGTGGAGGGAAAAAAGGGTGTAGGCTGGAGCTGCTTC
4	R_H2P2_prfH_KO_FRT	GGATGGCGTTTAATCGCCTTCCGGCAGTTTCATCCTTCATTATCCTCCTTAGTTCCTATTCC

*The following combination of primers were used for PCR amplification of the kanamycin cassette with FRT site, from pKD4 plasmid template, for which the PCR product was then transformed into electrocompetent cells expressing lambda-red recombineering system in cells harboring the pKD46 plasmid executed in both *E. coli BW25113* and *ATCC25922* strains for the knockout generation of *ΔrtcB2* (primers 1 and 2), *ΔprfH* (primers 3 and 4)and *ΔrtcB2-ΔprfH* (primers 1 and 4) genes.

Table S2. Primers used for PCR verification of successful knockouts.

Number	Primer name	Primer sequence
1	kanRt_fwd	TGCTCGACGTTGTCACTGAA
2	kanRt_rev	GATGTTTCGCTTGGTGGTCG
3	Fg_dinB_fwd	GGCAAATTTGGCCGCATTTTG
4	Rg_pepD_rev	GCATTTAGATCGTGAAGCGG
5	tyrTV_fwd	CACAGCTGAAGATATGATGCG
6	tyrtTV_rev	GGTGGTGGGGGAAGGATTAC

*In order to verify successful knockout strains, the following primers combinations were used 1-3, 2-4, and 3-4. When kanamycin was excised, the latter primers were utilized to score for the successful removal of the resistance cassette. Primers 5-6 were used to distinguish *E. coli BW25113* and *ATCC25922* strains.

Table S3. Primers used for cloning colicin E3 toxin using the NEBuilder® HiFi DNA Assembly kit.

Number	Primer name	Primer sequence
1	pASK_fwd	ATATCTTTGACCTGTGAAGTGAAAAATG
2	pASK_rev	CACCGCTCATTTTTTGCCCTCGTTATCTAG
3	Colicin E3_fwd	AGGGCAAAAAATGAGCGGTGGCGATGGAC
4	Colicin E3_rev	ACTTCACAGGTCAAAGATATTCTTGATATTTCGTTTCGGATCTG

Table S4. Primers used for qRT-PCR.

Number	Primer name	Primer sequence
1	EF-G_fwd	CGAAAGGCTACGAGTTCATC
2	EF-G_rev	CAGACGAATACCCATGTCTAC
3	PrfH_RT_fwd	GGCACTATTCAGTGGATTTG
4	PrfH_RT_rev	GCATCCGATTGTTCCTGCTC
5	RtcB2_RT_fwd	ATGGGCAAATATATTCGTCCC
6	RtcB2_RT_rev	CTGCATGTTGGGTAAATTTGC
